# Self-initiated feedback enhances reward positivity during motor learning in a standing weight-shifting task

**DOI:** 10.3389/fnhum.2026.1861427

**Published:** 2026-07-08

**Authors:** Ayumi Mochida, Tatsuya Iwabe, Akari Miyakawa, Susumu Yoshida

**Affiliations:** 1 Department of Physical Therapy, Graduate School of Rehabilitation Sciences, Health Sciences University of Hokkaido, Ishikari, Japan; 2 Department of Physical Therapy, School of Rehabilitation Sciences, Health Sciences University of Hokkaido, Ishikari, Japan

**Keywords:** agency, motor learning, postural control, reinforcement learning, reward positivity, weight-shifting task

## Abstract

**Background/Objectives:**

In motor learning, post-trial feedback provides critical information for interpreting action outcomes and adjusting subsequent motor output. One factor that may influence how feedback is processed is agency.

**Methods:**

Thirty-two healthy young adults performed a standing weight-shifting task in which they reached targets without an online center of pressure (COP) display. Feedback was displayed using colors and arrows. We compared self-initiated feedback (sFB), which participants obtained by pressing a button, with externally provided feedback (eFB), which was delivered by the experimenter. Electroencephalography (EEG) data were analyzed using a counterbalanced within-subject crossover design to compare feedback-related neural responses between conditions. Because the same task was performed in both phases, motor performance was compared between groups using data from the first phase only in order to avoid carryover effects. Reward positivity (RewP) was measured as a neural indicator of reward processing. Motor performance was evaluated as the distance error between the maximal COP position and the target location.

**Results:**

RewP was significantly larger in the sFB condition than in the eFB condition, suggesting enhanced reward processing under self-initiated feedback. During the training sessions, distance error decreased in both groups, indicating a learning effect. In the post-training test sessions, a significant main effect of group was observed. Exploratory *post hoc* comparisons suggested smaller distance errors in the sFB group during retention and transfer.

**Conclusion:**

In a standing weight-shifting task, self-initiated feedback enhanced RewP amplitude, which reflects reward processing, and improved retention of postural control ability. These findings suggest that obtaining feedback with a sense of agency influences neural reward processing and the retention of motor learning effects.

## 1 Introduction

Motor learning is thought to depend on at least two distinct learning signals: (i) sensory prediction error, which arises from the mismatch between predicted and actual sensory consequences, and (ii) reward prediction error, which reflects evaluative information about action success or failure (Izawa and Shadmehr, 2011; Wolpert et al., 2011). When sensory feedback is limited, reliance on reward prediction error becomes relatively greater (Izawa and Shadmehr, 2011). In redundant tasks where multiple movement solutions can lead to the same task outcome, sensory prediction error alone may be insufficient for identifying the optimal motor strategy. Under such conditions, evaluative information about success or failure may play an important role in behavioral updating (Wolpert et al., 2011).

Standing weight-shifting tasks may represent a motor learning context in which evaluative information about movement success or failure plays an especially important role. Although upper-limb reaching tasks have been widely used in motor learning research (Shadmehr et al., 2010; Wolpert et al., 2011), they typically allow learners to use relatively precise sensory error information, including hand or cursor position, to evaluate movement error and adjust subsequent motor commands (Izawa and Shadmehr, 2011; Wolpert et al., 2011). Thus, upper-limb reaching tasks often provide conditions under which sensory prediction error makes a major contribution to motor adaptation (Tseng et al., 2007; Izawa and Shadmehr, 2011). In contrast, in standing weight-shifting tasks, the center of mass (COM), the object being manipulated, cannot be directly perceived. To maintain upright posture, the COM must be controlled within the base of support (BOS), while the center of pressure (COP) serves as the mechanical output used to regulate balance (Winter, 1995; Horak, 2006). Moreover, because its state is derived from multiple sensory modalities (Horak, 2006), sensory error may be difficult to use as an effective signal for motor learning. Furthermore, voluntary COM shifting requires coordination across multiple joints within a multi-degree-of-freedom postural control system (Kilby et al., 2015). This task structure may make it difficult for learners to identify an optimal movement strategy based solely on sensory error information. Taken together, the limited availability of sensory error information and the need to coordinate multiple postural components suggest that evaluative information may be particularly important for motor learning in standing weight-shifting tasks.

In learning strategies based on reward prediction errors, action outcomes such as success or failure must be used to update subsequent behavior. Therefore, both the content of feedback and the way in which feedback is obtained may influence feedback processing. Previous studies have reported that self-controlled or self-initiated feedback can benefit motor learning (Chiviacowsky and Wulf, 2002; Grand et al., 2015). Specifically, whether feedback is obtained through one’s own action or presented externally may be important from the perspective of agency, which is defined as the extent to which outcomes are experienced as contingent on one’s own actions (Haggard, 2017). Agency is considered to involve multiple levels, ranging from the basic experience of performing an action to the higher-level experience of controlling one’s own actions (Synofzik et al., 2008; Moore, 2016). Thus, even simple actions such as pressing a button to obtain feedback may serve as an agency-related manipulation of feedback acquisition. Consistent with this view, reward-related event-related potential (ERP) studies have shown that neural responses vary according to the degree of personal involvement in choice or action initiation, suggesting that self-involvement in outcome acquisition may modulate reward processing (Yeung et al., 2005; Martin and Potts, 2011; Hassall et al., 2019). However, most of these studies have used simple choice tasks, and few have specifically investigated the act of receiving feedback itself in the context of motor learning using reward-related ERPs (Grand et al., 2015; Kim et al., 2019).

Among reward-related ERPs, the component observed over fronto-central scalp sites approximately 200–300 ms after feedback onset has been given several names, including feedback-related negativity (FRN) and reward positivity (RewP) (Proudfit, 2015; Krigolson, 2018). Although FRN remains widely used, RewP is increasingly preferred due to growing evidence that the gain-loss difference primarily reflects relative positivity following reward, rather than enhanced negativity following loss (Proudfit, 2015; Krigolson, 2018).

Accordingly, the present study used a standing weight-shifting task to compare a self-initiated feedback (sFB) condition, in which learners obtained feedback through their own action, with an externally provided feedback (eFB) condition, in which feedback was presented externally. We then examined whether self-involvement in obtaining feedback influenced neural indicators of reward processing and motor learning performance. We hypothesized that self-acquisition of feedback through a button press would function as a minimal form of agency, thereby enhancing reward-related feedback processing. Consequently, we expected that the sFB condition would elicit a larger RewP than the eFB condition and lead to better motor learning performance.

## 2 Materials and methods

### 2.1 Participants

A total of 32 healthy young adults (*M* = 20.9 years, SD = 1.01, range = 18–24, 11 females and 21 males) were recruited from undergraduate, graduate, and alumni groups at the Health Sciences University of Hokkaido. Eligibility criteria were (i) age 18–29 years and (ii) provision of written informed consent. Exclusion criteria included pain, neurological symptoms, dizziness, or limited range of joint motion that influenced ability to perform standing weight-shifting tasks. This study was approved by the Institutional Ethics Committee of Health Sciences University of Hokkaido (Approval No. 24R238258) and was conducted in accordance with the Declaration of Helsinki.

### 2.2 Experimental design

**Figure 1:**
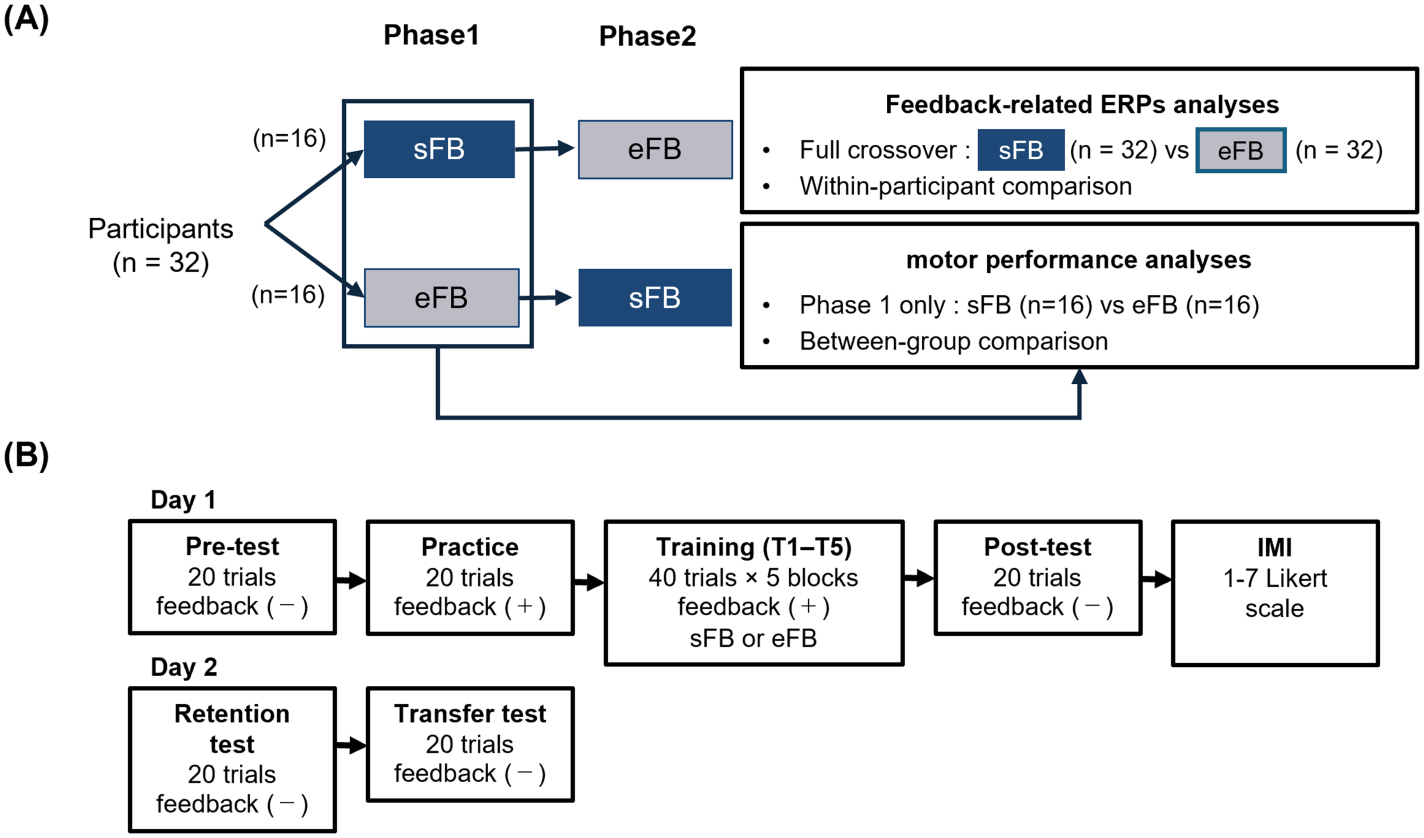
Overview of the experimental design and procedure. **(A)** Counterbalanced within-subject crossover design for comparisons between the sFB and eFB conditions. Motor performance was analyzed using phase 1 data only. **(B)** Study timeline for one phase. On day 1, participants completed the pre-test (20 trials, no feedback), practice (20 trials, feedback on all trials), training (T1–T5; 40 trials × 5 blocks = 200 trials; feedback on all trials), post-test (20 trials, no feedback), and the Intrinsic Motivation Inventory (IMI; 1 = strongly disagree to 7 = strongly agree). On Day 2, participants completed the retention test (20 trials, no feedback) and transfer test (20 trials, no feedback; different target distance).

Figure 1A shows the overall study design for the ERP and motor performance analyses. Because feedback-related ERP amplitudes, particularly the RewP, can vary across individuals (Hajcak et al., 2017; Faßbender et al., 2023), a counterbalanced, within-subject crossover design was adopted for electroencephalography (EEG) measures to minimize the influence of inter-individual variability and increase sensitivity to condition effects. EEG outcomes were therefore analyzed using within-subject comparisons across Phases 1 and 2. Participants were assigned to either the sFB group or the eFB group in Phase 1 using a minimization algorithm that balanced pre-test performance between groups. The interval between Phase 1 and Phase 2 was predefined as up to 1 month, a period over which test–retest reliability of ERP components has been reported to be maintained (Cassidy et al., 2012). The actual interval between the two phases was 10.4 ± 5.7 days, which fell within this range. Motor performance was analyzed using between-group comparisons based only on Phase 1 data, because participants performed the same task in both phases and Phase 2 data may have been affected by carryover effects.

Participants first completed 20 pre-test trials without feedback to assess baseline performance before training, followed by 20 practice trials with feedback to familiarize themselves with the task (Figure 1B). The training session comprised five blocks (T1–T5), each consisting of 40 trials, resulting in a total of 200 training trials. During training, feedback was provided on every trial. A post-test without feedback was administered immediately after training, and retention and transfer tests were conducted the next day, each consisting of 20 trials. In the transfer test, the target distance differed from that used in the other test sessions (Figure 1B).

After task completion, participants’ subjective experience of the task was assessed using the Intrinsic Motivation Inventory (IMI), a self-report questionnaire for intrinsic motivation (McAuley et al., 1989). In the present study, we used a shortened version of the IMI that was adapted based on a previous study (Badami et al., 2011). This shortened version comprised nine items across three subscales of the IMI (interest/enjoyment, perceived competence, and effort/importance), with each subscale including three items rated on a 7-point Likert scale. Internal consistency was assessed using Cronbach’s alpha, which indicated acceptable reliability for the three subscales: interest/enjoyment (*α* = 0.83), perceived competence (*α* = 0.84), and effort/importance (*α* = 0.80). The total score across all items was used as a composite measure of intrinsic motivation (Badami et al., 2011). Possible scores ranged from 9 to 63, with higher scores indicating greater intrinsic motivation.

### 2.3 Task and apparatus

**Figure 2:**
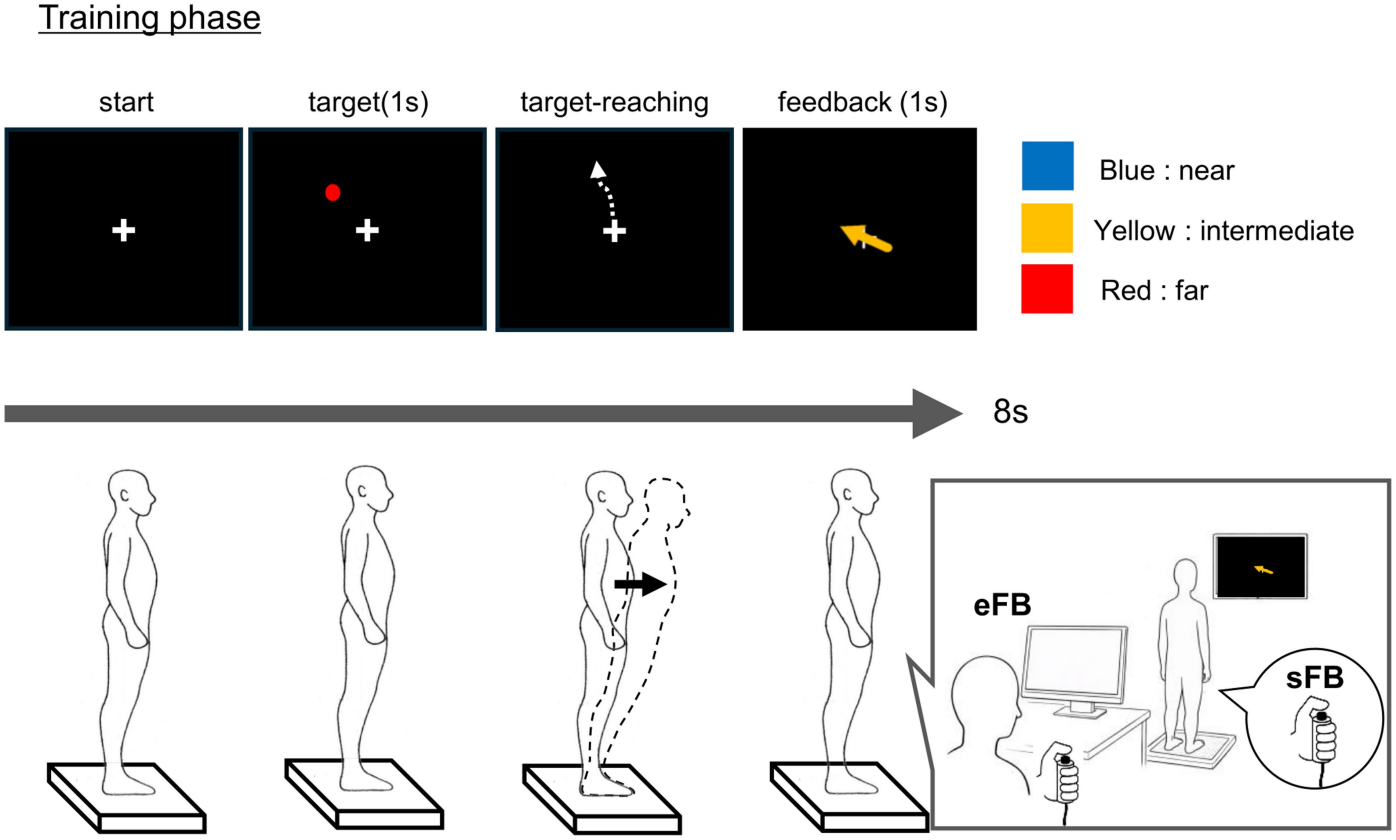
Details of the standing weight-shifting task. Following target presentation for 1 s, participants shifted their COP toward the target position and returned to the starting position. Feedback indicating direction (arrow) and distance (blue/yellow/red) was then presented for 1 s. The feedback acquisition condition was either self-initiated (sFB) or externally provided (eFB).

Figure 2 illustrates the standing weight-shifting task used in this study. Participants stood barefoot on the force plate with a stance width of 10 cm, maintaining an upright posture with both arms hanging at their sides. They were instructed to shift their COP toward the target position and then immediately return to the central position without visual feedback of their own COP. Visual targets and feedback were presented on a 27″ LCD monitor positioned 140 cm in front of the center of the force plate. The monitor height was adjusted so that the fixation point was at eye level for each participant.

COP was measured using a custom-built force plate consisting of four load cells (LTZ-100KA-P, Kyowa Electronic Instruments Co., Ltd., Japan). The signals were amplified using a signal conditioner (CDV-90A, Kyowa Electronic Instruments Co., Ltd., Japan), digitized via an A/D converter (USB-6215, National Instruments), and transmitted to a laptop computer for target and feedback presentation. In parallel, the amplified analog signals were split and recorded through a second A/D interface (Micro1401-4 with Spike2, CED) for offline analyses. Targets and feedback were generated using custom software (PERITEC Corporation, Japan) implemented in LabVIEW 2024 (National Instruments, United States).

The target distance was set to 20% of foot length in the pre-test, post-test, and retention test, and a distance of 40% of foot length in the transfer test. The target consisted of a white circle (1 cm in diameter) presented on a black background for 1 s at 0 °, ±20 °, or ±45 ° (+ right, − left) along a frontal arc with a radius corresponding to the target distance. Motor performance was quantified as distance error (mm), defined as the Euclidean distance between the target position and the COP position at the time when the COP *y*-coordinate reached its maximum value during the reaching movement. Smaller values indicated better performance. Feedback was provided after a button press using color and arrow indicators based on the magnitude and direction of the distance error.

In the sFB condition, participants obtained feedback by pressing a button. To reduce the influence of movement-related cortical activity associated with the button press on feedback-locked ERPs, feedback was presented 0.5 s after the button press in both conditions. In the eFB condition, feedback was presented externally at a time determined by the experimenter. Feedback onset latency was calculated for each trial as the time interval between the peak of the COP y-coordinate during the weight-shifting movement toward the target position and the onset of feedback. Across participants, the median feedback onset latency was 2.11 s (interquartile range (IQR) = 0.39 s) in the sFB condition and 2.46 s (IQR = 0.34 s) in the eFB condition.

To facilitate motor learning, feedback was presented in three graded outcome levels: blue for positive feedback (error ≤ success threshold), yellow for intermediate feedback (success threshold < error ≤ intermediate boundary), and red for negative feedback (error > intermediate boundary). Distance error relative to the target was averaged within each block after excluding outliers (±2 standard deviations (SD)). The blue threshold corresponded to the mean − SD, the yellow threshold was defined as the blue threshold +5 mm, and values exceeding this threshold were classified as red. Thresholds calculated from the preceding block were used as adaptive success criteria for the subsequent block. For the first block, the value obtained during the practice session was used. Participants were informed of the meaning of each color before the experiment.

### 2.4 EEG preprocessing

EEG data were recorded using an active electrode cap (actiCAP slim; Brain Products GmbH) from 31 scalp sites positioned according to the extended international 10–20 system (Fp1, Fz, F3, F7, FT9, FC5, FC1, C3, T7, TP9, CP5, CP1, Pz, P3, P7, O1, O2, P4, P8, TP10, CP6, CP2, Cz, C4, T8, FT10, FC6, FC2, F4, F8, Fp2). Signals were amplified with a BrainAmp DC amplifier (Brain Products GmbH) and digitized at 1000 Hz. The EEG was referenced online to linked earlobes (A1/A2), with the ground electrode placed at Fpz. Electrode impedances were continuously monitored and maintained below 10 kΩ. To monitor blink-related activity, vertical electrooculography (EOG) was recorded using a bipolar montage with electrodes placed above and below the right eye.

Offline preprocessing was performed using BrainVision Analyzer 2.3 (Brain Products GmbH). Continuous data were band-pass filtered (0.1–30 Hz) and notch-filtered at 50 Hz. Independent component analysis was applied to long epochs (−1000 to +2000 ms relative to feedback onset) to remove ocular artifacts, based on visual inspection of IC scalp topographies (Colino et al., 2020). The corrected data were then re-segmented into feedback-locked epochs from −200 to +600 ms and baseline-corrected using the pre-feedback interval (−200 to 0 ms) (Colino et al., 2020). Because the RewP is typically observed over a broad fronto-central scalp distribution, it is commonly quantified at electrodes such as Fz, FCz, and Cz (Sambrook and Goslin, 2015; Glazer et al., 2018). As FCz was not included in the present montage, RewP was analyzed at Fz and Cz. Artifact rejection was performed in two steps using BrainVision Analyzer’s automatic artifact rejection functions. First, automatic segment rejection was applied to the fronto-central channels of interest (Fz and Cz) using the gradient criterion (10 μV/ms) and the max–min criterion (maximum–minimum difference within the epoch: 100 μV) (Colino et al., 2020). Subsequently, all remaining epochs were visually inspected to confirm artifact rejection and ensure that residual artifacts did not contaminate the average ERPs. Datasets with fewer than 20 artifact-free epochs for the relevant feedback conditions were excluded from ERP analyses. As a supplementary ERP measure to capture later-stage feedback processing, P300 was quantified at Pz (Polich, 2007).

### 2.5 ERP quantification

The primary ERP outcome measure was RewP, which was derived from feedback-locked ERPs at electrodes Fz and Cz. ERPs were averaged separately for positive (Blue) and negative (Red) feedback, and a difference waveform (Blue − Red) was computed. Yellow feedback was excluded from the RewP analysis because the primary contrast of interest was between positive and negative outcomes. RewP amplitude was quantified as the mean voltage within a 200–300 ms window after feedback onset, consistent with the typical time range of the RewP component (Glazer et al., 2018). We used mean amplitude rather than peak amplitude because it is less sensitive to noise and provides a more robust estimate within a predefined time window (Luck, 2005; Clayson et al., 2013). P300 amplitude was quantified at Pz separately for red and blue feedback. Mean amplitude was calculated within a 300–500 ms window, based on standard definitions of the P300 component (Polich, 2007).

### 2.6 Statistical analysis

*A priori* sample size calculation was performed using G*Power (version 3.1.9.7) for the within-subject comparison of RewP between the sFB and eFB conditions. Assuming an effect size of *d* = 0.60, *α* = 0.05, and power (1 − *β*) = 0.80, the required sample size was 24. The assumed effect size was conservatively set based on the effect size reported in a comparable study (*d* = 0.87; Grand et al., 2015), while also considering the design characteristics of the present study. Because RewP was the primary outcome and some EEG datasets could be excluded due to artifacts or insufficient valid epochs, the target sample size was set at 32. All analyses were conducted using IBM SPSS Statistics (version 30; IBM Corp.).

RewP was analyzed using repeated-measures analysis of variance (ANOVA) with the feedback condition and electrode site as within-subject factors. P300 was analyzed using repeated-measures ANOVA with feedback condition and color as within-subject factors.

Motor performance was analyzed using linear mixed-effects models. For the test sessions, distance error was examined with pre-test performance as a covariate and with group (sFB vs. eFB), session (post-test, retention test, transfer test), and their interaction included as fixed effects. When a significant main effect of group was observed, Bonferroni-adjusted between-group comparisons were performed for each session. For the training sessions, distance error was examined with group (sFB vs. eFB), block (T1–T5), and their interaction included as fixed effects. Total IMI scores were compared using the Wilcoxon signed-rank test. The level of statistical significance for all tests was set at *p* < 0.05.

## 3 Results

### 3.1 Feedback-related ERP results

**Figure 3:**
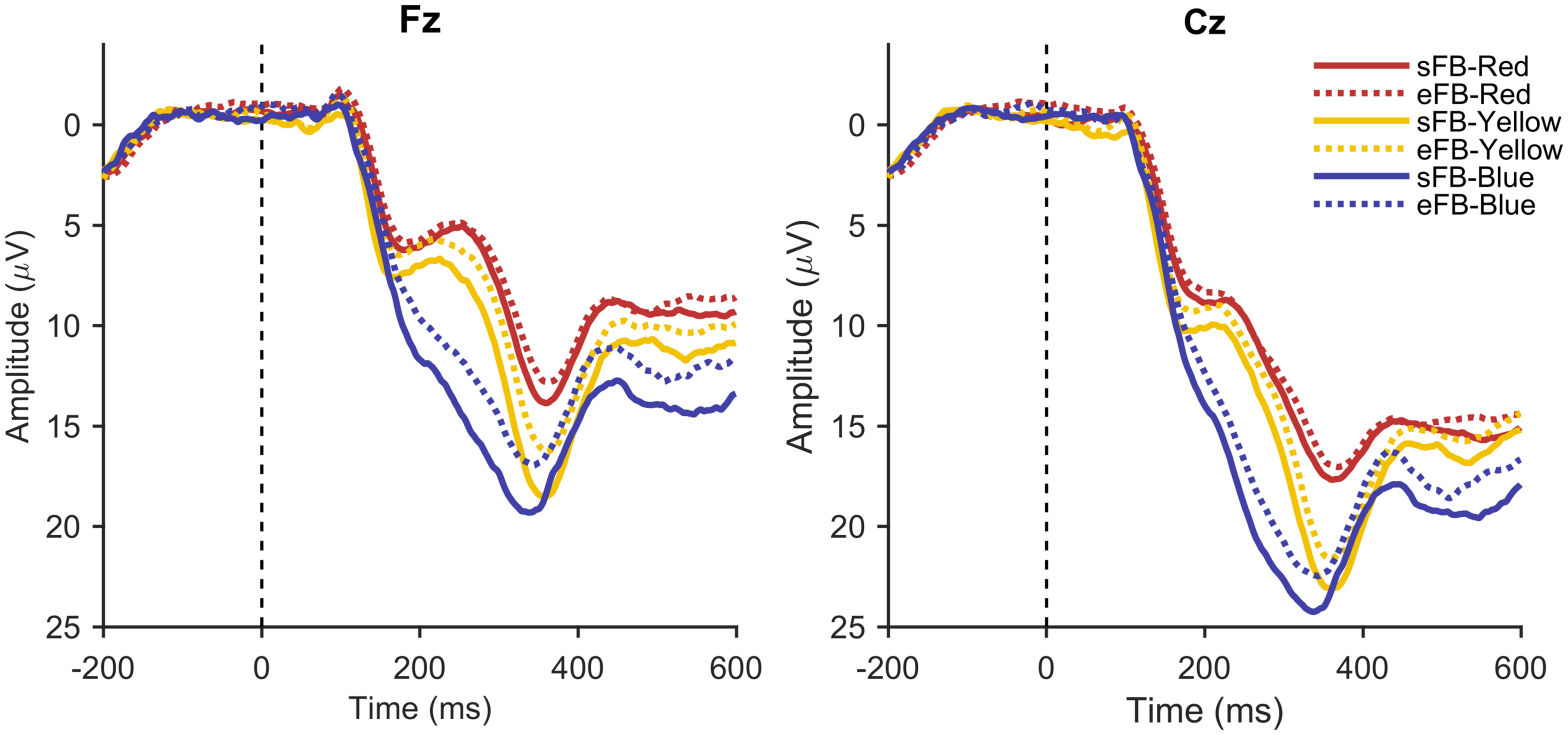
Grand-average feedback-locked ERPs at Fz and Cz for each feedback color. Waveforms are time-locked to feedback onset (0 ms) and plotted from −200 to 600 ms. For each feedback color (blue, yellow, red), solid lines indicate the sFB condition and dashed lines indicate the eFB condition (*N* = 31).

ERP analyses were conducted on data from 31 participants, after excluding one dataset due to an EEG recording malfunction. Table 1 shows the number of epochs for each feedback color included in the averaging. No significant differences between the sFB and eFB conditions were observed for the frequency of any feedback color: blue (*Z* = −0.49, *p* = 0.624, *r* = 0.09), yellow (*Z* = −0.14, *p* = 0.891, *r* = 0.03), and red (*Z* = −0.14, *p* = 0.891, *r* = 0.03). The grand-average feedback-locked ERPs at Fz and Cz for each feedback color (blue, yellow, and red) in the sFB and eFB conditions are shown in Figure 3.

**Table 1:** Number of artifact-free epochs per feedback color.

Feedback color	sFB epochs	eFB epochs
Blue	40.65 ± 13.22 (22–82)	41.42 ± 10.75 (24–64)
Yellow	56.71 ± 14.57 (31–86)	53.26 ± 14.45 (23–81)
Red	91.00 ± 17.55 (58–123)	90.90 ± 19.71 (58–131)

Values are mean ± SD (min–max).

**Figure 4:**
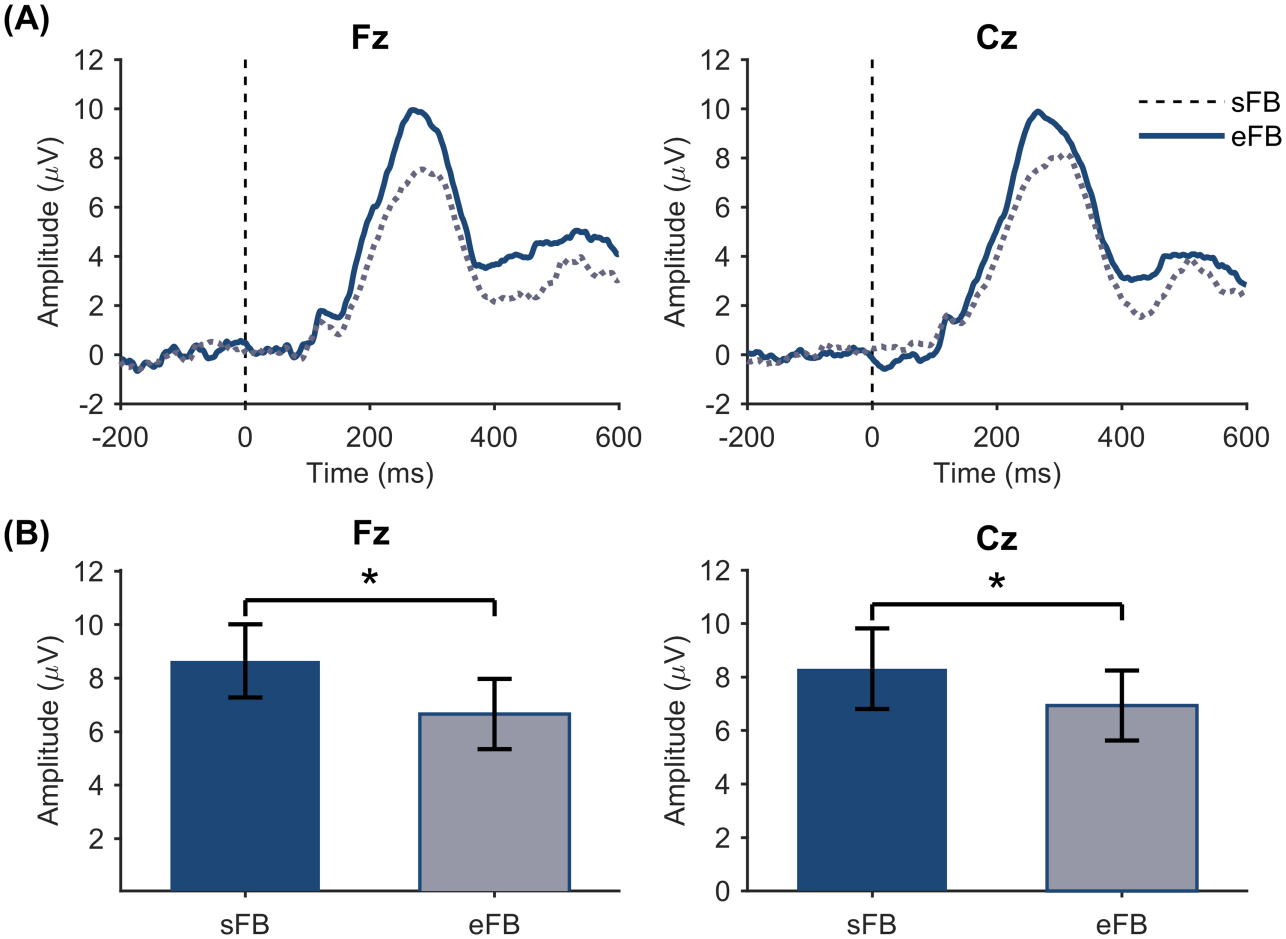
Grand-average RewP and mean amplitude. **(A)** Grand-average RewP (Blue–Red) waveforms at Fz and Cz, time-locked to feedback onset (0 ms) and plotted from −200 to 600 ms. **(B)** Mean RewP amplitude in the 200–300 ms window for the sFB and eFB conditions at each electrode. Bars represent condition means, and error bars indicate 95% CIs.

Figure 4A shows the grand-average RewP (Blue − Red) waveforms at Fz and Cz, and Figure 4B shows the mean RewP amplitudes in the 200–300 ms window after feedback onset. ANOVA revealed a significant main effect of condition (*F* (1, 30) = 7.11, *p* = 0.012), indicating that RewP was larger in the sFB condition (EMM = 8.41 μV, SE = 0.69, 95% CI [6.99, 9.82]) than in the eFB condition (EMM = 6.53 μV, SE = 0.57, 95% CI [5.37, 7.70]). Neither the main effect of electrode (*F* (1, 30) = 0.21, *p* = 0.651) nor the condition × electrode interaction (*F* (1, 30) = 0.50, *p* = 0.484) was significant. Follow-up comparisons indicated that RewP was larger in the sFB condition than in the eFB condition at both Fz (*p* = 0.010) and Cz (*p* = 0.036).

**Figure 5:**
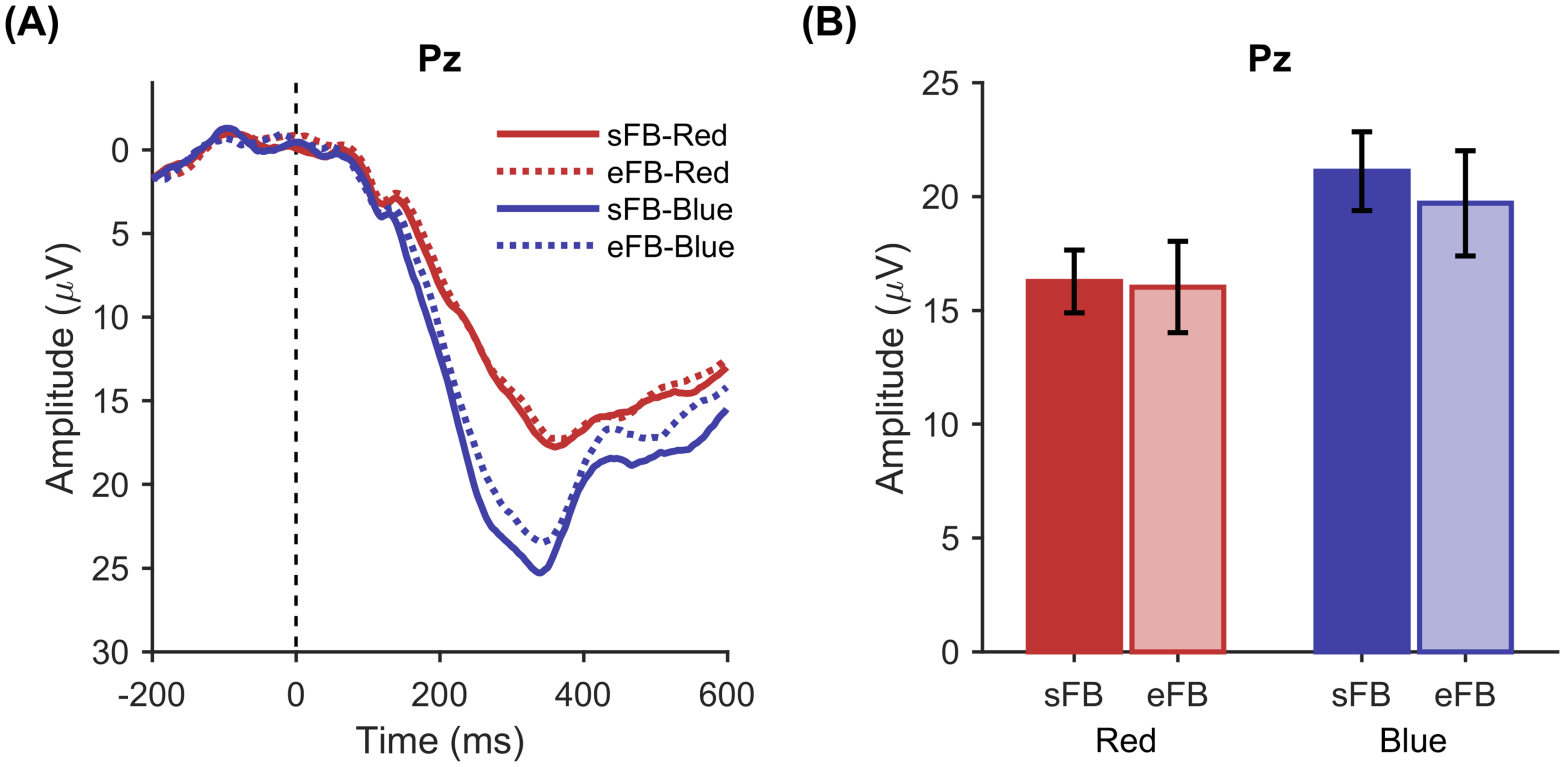
P300 at Pz for red and blue feedback. **(A)** Grand-average feedback-locked ERPs at Pz for red and blue feedback in the sFB and eFB conditions, time-locked to feedback onset (0 ms) and plotted from −200 to 600 ms. **(B)** Mean P300 amplitude at Pz, computed as the mean voltage in the 300–500 ms window for each condition and feedback color. Bars represent condition means, and error bars indicate 95% CIs.

P300 amplitude at Pz was quantified separately for red and blue feedback as the mean voltage in the 300–500 ms window after feedback onset. Figure 5A shows the grand-average feedback-locked ERPs at Pz for red and blue feedback in the sFB and eFB conditions, and Figure 5B summarizes the mean P300 amplitudes with 95% CIs. ANOVA revealed a significant main effect of color (*F* (1, 30) = 68.79, *p* < 0.001). In contrast, neither the main effect of condition (*F* (1, 30) = 1.12, *p* = 0.299) nor the condition × color interaction (*F* (1, 30) = 1.57, *p* = 0.219) was significant. Collapsed across conditions, P300 amplitude was larger for blue feedback (EMM = 20.41 μV, SE = 0.86, 95% CI [18.66, 22.16]) than for red feedback (EMM = 16.15 μV, SE = 0.75, 95% CI [14.62, 17.68]).

### 3.2 Motor performance and motivational results

**Figure 6:**
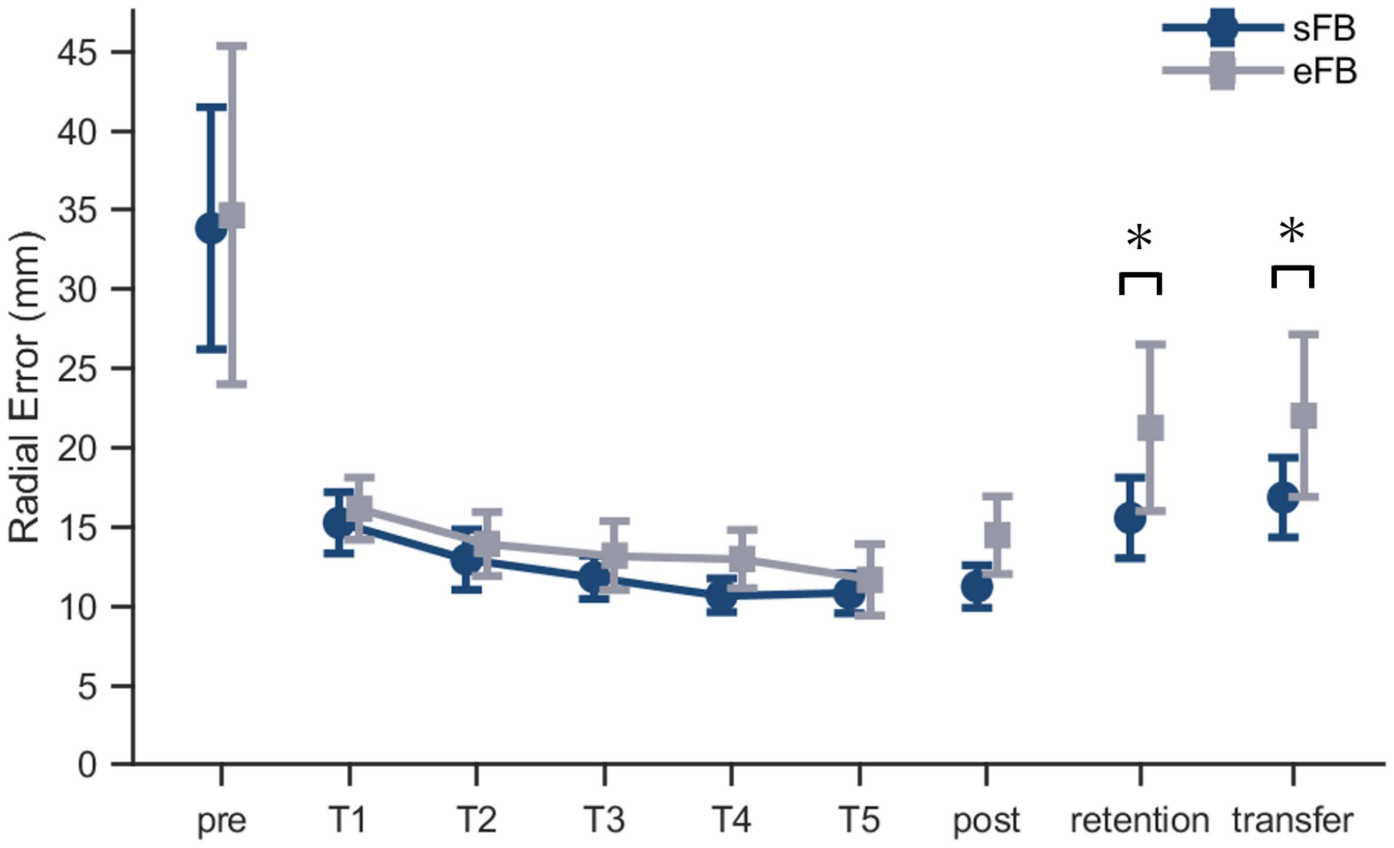
Motor performance (distance error) across training blocks (T1–T5) and test sessions (pre-test, post-test, retention, and transfer). Values represent observed means calculated after excluding outlier trials (mean ± 2 SD) within each session or block. Error bars indicate 95% confidence intervals.

A significant main effect of group was observed for distance error (*F* (1, 29) = 6.11, *p* = 0.020), indicating smaller distance errors in the sFB group than in the eFB group. The group × test session interaction was not significant (*F* (2, 60) = 0.81, *p* = 0.452). Exploratory between-group comparisons for each test session showed significantly smaller distance errors in the sFB group than in the eFB group in the retention test (difference = −5.57, *p* = 0.014) and transfer test (difference = −5.05, *p* = 0.025). No significant between-group differences were observed in the post-test (difference = −3.13, *p* = 0.160; Figure 6).

A significant main effect of block was observed during training, indicating that distance error decreased across blocks in both groups (*F* (4, 120) = 17.64, *p* < 0.001). Neither the main effect of group (*F* (1, 30) = 1.80, *p* = 0.189) nor the group × block interaction (*F* (4, 120) = 0.52, *p* = 0.719) was significant (Figure 6).

Total IMI scores did not differ significantly between the sFB and eFB conditions (Wilcoxon signed-rank test, *Z* = −0.44, *p* = 0.657). The median [IQR] composite score was 48.0 [41.25–50.75] in the sFB condition and 44.5 [41.0–52.0] in the eFB condition.

## 4 Discussion

### 4.1 Summary of main findings

This study used a standing weight-shifting task to compare sFB and eFB conditions and examine how self-involvement in obtaining feedback influences neural indicators of feedback processing and motor learning performance. We found that RewP was significantly larger in the sFB condition than in the eFB condition at both Fz and Cz. In contrast, P300 showed only a main effect of feedback color and did not differ between conditions. Distance error was smaller overall in the sFB group than in the eFB group across the post-practice test sessions, although the group × test session interaction was not significant. During training, distance error decreased across blocks in both groups, with no significant between-group difference. IMI scores also did not differ significantly between conditions.

### 4.2 Effects of self-involvement in obtaining feedback on ERPs

The presence of RewP in both conditions suggests that the color- and arrow-based feedback used in our task was processed as rewarding. Importantly, RewP was significantly larger in the sFB condition than in the eFB condition. These findings are consistent with previous studies that manipulated agency (Yeung et al., 2005; Hassall et al., 2019). RewP is a feedback-locked ERP component that is associated with activity in reward-related regions of the medial frontal cortex, including the anterior cingulate cortex (ACC) (Becker et al., 2014). These regions are thought to process reward and error information in ways that support the identification of appropriate behaviors and contribute to subsequent behavioral adjustment (Holroyd and Coles, 2002). Reward prediction errors, which indicate whether an outcome is better or worse than expected, have been described as reinforcement-learning signals conveyed to the ACC via the mesencephalic dopamine system and used to guide adaptive behavioral modification (Holroyd and Coles, 2002). Accordingly, receiving feedback through one’s own action may have enhanced the processing of outcome information as a basis for selecting appropriate behavior and subsequent behavioral adjustment.

Because the feedback content was kept comparable between conditions, the present design allowed us to examine whether self-initiated feedback acquisition, rather than feedback content or feedback scheduling, modulated reward-related neural processing. One possible explanation for the enhanced RewP is agency. Haggard defined agency as the feeling of generating and controlling external events through one’s own actions (Haggard, 2017). Previous studies have shown that the RewP, as a reward-related neural response, is enhanced when outcomes are linked to one’s own choices or actions (Hassall et al., 2019; Zheng et al., 2020). Although the present study did not manipulate choice, it did include an aspect of motor agency because feedback in the sFB condition was initiated by the participant’s own button press (Kaiser et al., 2021). Consequently, outcomes in the sFB condition may have been more readily processed as consequences of one’s own actions, contributing to the enhanced RewP. Although a previous gambling-task study suggested that a simple button press does not enhance RewP without choice (Hassall et al., 2019), the present findings suggest that, in a standing weight-shifting task where evaluative feedback is important for learning, even a minimal agency-related action to obtain feedback may contribute to enhanced RewP. However, because subjective sense of agency was not directly measured, related processes such as action–outcome linkage, self-relevance of feedback, attentional engagement, or perceived control over feedback presentation may also have contributed to the enhanced RewP.

In contrast, P300 did not differ significantly between the sFB and eFB conditions. Previous studies suggest that feedback-related P300 reflects later attentional processing of outcomes as well as motivation toward the task (Yeung et al., 2005; Hassall et al., 2019; Zheng et al., 2020). In the present study, however, task characteristics such as feedback frequency and task difficulty were the same across conditions, and IMI scores did not differ between conditions. These findings suggest that attention and motivation may not have differed enough between conditions to generate a detectable difference in P300. The main effect of color likely reflects the lower frequency of blue feedback relative to red feedback. Thus, the differences in P300 in the present study may reflect stimulus rarity more strongly than the positive or negative nature of the outcome itself (Luck, 2005).

### 4.3 Behavioral implications for motor learning

Both groups improved through practice, suggesting that participants learned the weight-shifting task during training. In the present task, participants had to explore weight-shifting strategies based on bodily sensations, without direct visual confirmation of their COP position, and then interpret outcome information after each trial through feedback presented by colors and arrows. In this context, learning may be more appropriately understood as a process of updating behavioral strategies based on movement outcomes, rather than online correction based on continuous visual error signals (Izawa and Shadmehr, 2011). This learning process may also have a reinforcement learning-like aspect, in which behavioral strategies are updated on the basis of reward prediction error (Barto, 1994; Sutton and Barto, 2018).

Although no interaction was observed, a significant main effect of group was observed across the post-practice test sessions, indicating that the sFB group showed smaller distance errors than the eFB group overall. Exploratory *post hoc* comparisons revealed significant differences in the retention and transfer tests. These session-specific findings should be interpreted cautiously; nevertheless, they suggest that self-initiated feedback acquisition may facilitate the maintenance of learned performance and its application to a modified task context. This pattern may reflect both reward-based reinforcement learning processes and agency-related influences. Previous studies have proposed several mechanisms by which reward- or reinforcement-based learning may facilitate retention. In upper-limb visuomotor adaptation, reward-based feedback has been suggested to strengthen the memory trace of a newly learned visuomotor transformation, possibly through reward-related dopaminergic signals to M1 (Galea et al., 2015). In gait learning, a reinforcement-learning approach has been proposed to improve retention by encouraging self-guided exploration of rewarding actions and by reducing reliance on directional supervisory feedback (Hasson et al., 2015). In the present study, self-initiated feedback may have similarly encouraged active processing of action outcomes, and the enhanced RewP observed in the sFB condition is consistent with this interpretation.

In addition, agency and beliefs about whether one’s own actions caused an outcome have been reported to influence reward-related learning and behavioral updating through striatal processes (Render and Jansen, 2019; Dorfman et al., 2021). Because RewP is thought to reflect reward-related processing in medial frontal regions including the ACC (Holroyd and Coles, 2002), this interpretation is consistent with the neural findings described in Section 4.2 where RewP was enhanced in the sFB condition. Obtaining feedback through one’s own action may have promoted deeper processing of outcome information, thereby contributing to the post-practice retention observed in the sFB group.

Learning transfer is more likely to occur when the transfer task is more similar to the original learning task (Shadmehr, 2004). Because the transfer task in the present study differed from the learning task only in the target distance, while target direction remained unchanged, it is possible that the same learning processes underlying retention contributed to the transfer performance.

### 4.4 Limitations and future directions

This study is subject to several limitations. First, the influence of movement-related cortical potentials on the ERP findings cannot be fully ruled out. To reduce this possibility, we inserted a 0.5 s delay between the button press and feedback onset. However, because of the task characteristics, the EEG signal preceding the button press was likely to contain artifacts associated with postural control. This issue should therefore be considered when interpreting the ERP findings.

Second, the neural and behavioral outcomes were evaluated using different analytical frameworks: ERP measures were analyzed using a crossover design, whereas motor performance was assessed using only Phase 1 data to avoid carryover effects. As a result, the relationship between feedback-related neural responses and post-practice motor performance could not be directly examined under identical experimental conditions. Future studies could address this issue by adopting designs that allow neural and behavioral measures to be evaluated more directly within the same analytical framework.

Finally, the behavioral analysis may have had limited sensitivity to detect subtle effects across test sessions. Because the behavioral analysis was restricted to Phase 1 data to minimize potential carryover effects, the effective sample size was relatively small. This may have reduced the statistical power to detect a feedback condition × test session interaction or subtle session-specific differences. Therefore, the absence of significant interaction should be interpreted with caution. Although distance error was smaller overall in the sFB group across the post-practice tests, the session-specific behavioral findings at retention and transfer should be interpreted with caution. Further studies using larger samples are needed to clarify the behavioral effects of feedback acquisition methods in the post-practice stage.

## 5 Conclusion

The present findings suggest that, in a standing weight-shifting motor learning task, obtaining feedback through one’s own action during the acquisition phase may enhance outcome-related neural processing and facilitate motor learning after practice. Specifically, we found that self-initiated feedback enhanced RewP amplitudes, suggesting enhanced reward-related processing, and was associated with better retention of postural control performance. Reward-based modulation of motor learning has been proposed as a potential avenue for improving upper-limb rehabilitation after stroke (Quattrocchi et al., 2017; Palidis et al., 2025). Although the present study was conducted in healthy young adults, our findings raise the possibility that self-initiated acquisition of feedback may further enhance the effectiveness of reward-based feedback in applied motor learning contexts. We also examined feedback-related neural responses and motor learning performance within the same experiment, which is relatively uncommon in motor learning research. However, as the relationship between these neural and behavioral measures was not directly tested, the findings do not allow us to make firm conclusions about whether neural enhancement contributed to the behavioral advantage. Future studies are needed to directly examine how feedback-related neural responses are linked to later motor learning outcomes.

## Data Availability

The raw data supporting the conclusions of this article will be made available by the authors, without undue reservation.
